# In patients with metastatic breast cancer the identification of circulating tumor cells in epithelial-to-mesenchymal transition is associated with a poor prognosis

**DOI:** 10.1186/s13058-016-0687-3

**Published:** 2016-03-09

**Authors:** Michela Bulfoni, Lorenzo Gerratana, Fabio Del Ben, Stefania Marzinotto, Marisa Sorrentino, Matteo Turetta, Giacinto Scoles, Barbara Toffoletto, Miriam Isola, Carlo Alberto Beltrami, Carla Di Loreto, Antonio Paolo Beltrami, Fabio Puglisi, Daniela Cesselli

**Affiliations:** Department of Medical and Biological Sciences, University of Udine, P.le Kolbe 4, 33100 Udine, Italy; Department of Oncology, University Hospital of Udine, P.le S.Maria della Misericordia 15, 33100 Udine, Italy; Institute of Pathology, University Hospital of Udine, P.le S.Maria della Misericordia 15, 33100 Udine, Italy

## Abstract

**Background:**

Although recent models suggest that the detection of Circulating Tumor Cells (CTC) in epithelial-to-mesenchymal transition (EM CTC) might be related to disease progression in metastatic breast cancer (MBC) patients, current detection methods are not efficient in identifying this subpopulation of cells. Furthermore, the possible association of EM CTC with both clinicopathological features and prognosis of MBC patients has still to be demonstrated. Aims of this study were: first, to optimize a DEPArray-based protocol meant to identify, quantify and sort single, viable EM CTC and, subsequently, to test the association of EM CTC frequency with clinical data.

**Methods:**

This prospective observational study enrolled 56 MBC patients regardless of the line of treatment. Blood samples, depleted of CD45^pos^ leukocytes, were stained with an antibody cocktail recognizing both epithelial and mesenchymal markers. Four CD45^neg^ cell subpopulations were identified: cells expressing only epithelial markers (E CTC), cells co-expressing epithelial and mesenchymal markers (EM CTC), cells expressing only mesenchymal markers (MES) and cells negative for every tested marker (NEG). CTC subpopulations were quantified as both absolute cell count and relative frequency. The association of CTC subpopulations with clinicopathological features, progression free survival (PFS), and overall survival (OS) was explored by Wilcoxon-Mann-Whitney test and Univariate Cox Regression Analysis, respectively.

**Results:**

By employing the DEPArray-based strategy, we were able to assess the presence of cells pertaining to the above-described classes in every MBC patient. We observed a significant association between specific CD45^neg^ subpopulations and tumor subtypes (e.g. NEG and triple negative), proliferation (NEG and Ki67 expression) and sites of metastatic spread (e.g. E CTC and bone; NEG and brain). Importantly, the fraction of CD45^neg^ cells co-expressing epithelial and mesenchymal markers (EM CTC) was significantly associated with poorer PFS and OS, computed, this latter, both from the diagnosis of a stage IV disease and from the initial CTC assessment.

**Conclusion:**

This study suggests the importance of dissecting the heterogeneity of CTC in MBC. Precise characterization of CTC could help in estimating both metastatization pattern and outcome, driving clinical decision-making and surveillance strategies.

**Electronic supplementary material:**

The online version of this article (doi:10.1186/s13058-016-0687-3) contains supplementary material, which is available to authorized users.

## Background

Circulating tumor cells (CTC) are rare cells shed into the bloodstream from primary tumors and metastases [1]. Since these latter represent the major cause of cancer-associated mortality [2], CTC isolation and characterization is one of the most active areas of translational cancer research [1]. In fact, CTC might represent an active source of metastatic spread from a primary tumor to secondary lesions [3, 4], and their role as a prognostic biomarker has been robustly demonstrated both in primary and metastatic cancer [5–9].

Moreover, detection and enumeration of CTC could serve as an early marker of response to systemic therapy, whereas the molecular characterization of CTC could lead to individualized targeted treatments, possibly sparing patients unnecessary and ineffective therapies [10].

Current models suggest that the invasive phenotype of breast cancers is mostly associated with an epithelial-to-mesenchymal transition (EMT) [11]. This process leads to the expression of mesenchymal markers on tumor cells, which is paralleled by an increase in the migration and invasion properties of tumor cells, as well as in their resistance to apoptosis and ability to evade the immune response [11]. The detection of CTC that express either mesenchymal and epithelial mRNAs or only mesenchymal mRNAs could therefore be related, in metastatic breast cancer (MBC) patients, to disease progression [12]. However, existing detection methods are not efficient in identifying CTC in EMT. In fact, the only Food and Drug Administration (FDA)-approved device to detect CTC, the CellSearch System (Veridex, Warren, NJ, USA), allows counting only epithelial cell adhesion molecule (EpCAM)-positive epithelial CTC. Moreover, this device does not allow harvesting viable CTC suitable for downstream analyses. For this reason, in the last years several innovative strategies to enrich, detect, count, and/or molecularly characterize CTC have been developed [13]. However, for most of these a clinical validation is still missing [14].

DEPArray (Silicon Biosystems, Bologna, Italy) is a dielectrophoresis-based platform able to handle a relatively small number of cells. The device is aimed at analyzing and sorting single, viable, rare cells thanks to an image-based selection process and to the entrapment of cells inside dielectrophoretic cages. Selected cells can be individually moved by software-controlled modulation of electrical fields and ultimately recovered for downstream molecular analyses [15].

The main objectives of our study were: to develop a novel strategy to enrich blood samples in CTC, independently from the expression of epithelial markers; to take advantage of the DEPArray system to identify and sort, based on a multiparametric fluorescence analysis, single, viable epithelial-like CTC as well as CTC in EMT; to explore, in a prospective observational case study including 56 patients with MBC, the association between clinicopathological features, CTC number, and distribution of CTC subpopulations; and, finally, to provide evidence of the possible prognostic role of the enumeration of CTC in EMT.

## Methods

### Patient population, ethics, consent, and permissions

Our prospective observational study was approved by the Local Ethics Committee (decision No. 152/2011/Sper) and, subsequently, by the Regional Ethics Committee (amendment No. 178/2014/Em). Fifty-six patients were enrolled by the Department of Oncology, University Hospital of Udine, Italy. Of these, 47 blood samples were analyzed for the presence of CTC by the Department of Pathology, University Hospital of Udine, Italy. Nine patients were excluded from the analysis because the volume of sampled blood was inadequate (*n* = 1), the timing of blood sampling was incorrect (*n* = 3), or the samples were employed to optimize the technical procedure (*n* = 5). All patients gave their written informed consent before their enrollment.

### Patient enrollment and follow-up

The eligibility criteria for patient recruitment were as follows: female, adult (≥ 18 years) patients with measurable MBC, at the start of a new systemic therapy, without limits to number and kind of previous therapies (hormone therapy, chemotherapy, targeted therapy), and Eastern Cooperative Oncology Group performance status (ECOG PS) score ≤ 2. A histological sample representative of the primary tumor had to be available.

Before starting a new treatment, patients underwent baseline blood sampling for CTC evaluation (see later), and standard clinical studies.

Standard Response Evaluation Criteria In Solid Tumors (RECIST) criteria were used to determine patients’ responses to treatment [16].

An expert pathologist reviewed the biopsies of every enrolled patient, defining the tumor type following the WHO classification of breast cancer [17], grading as in [18], and tumor subtype as in [19].

### Sampling of biologic material

Blood samples of 15 ml of blood were collected from 56 MBC patients and 27 healthy donors. Ethylenediamine tetraacetate was used as anticoagulant. The samples were processed within 4 hours from blood withdrawal. Healthy control blood samples were collected from female blood donors aged 20–60 years.

### Flow cytometry

Epithelial-like (ER^+^/PR^+^/HER2^−^ MCF-7; ATCC, Teddington, Middlesex, UK) and mesenchymal-like (triple-negative MDA-MB231; ATCC, ​Teddington, Middlesex, UK) breast cancer cell lines were tested by flow cytometry, to determine their immunophenotype, employing monoclonal antibodies labeled with phycoerythrin (PE), fluorescein isothiocyanate (FITC), or allophycocyanin (APC) and directed against: CD10 (BD Biosciences, San Jose, CA, USA), CD29 (clone TS2/16; eBioscience, San Diego, CA, USA), CD44 (clone IM7; Biolegend, San Diego, CA, USA), CD45 (BD Pharmingen, BD Bioscience, San Jose, CA, USA / Milenyi Biotec, Calderara di Reno, BO, Italy), CD49a (clone TS2/7; Biolegend), CD49b (BD Pharmingen), CD49d (clone 9F10; Biolegend, San Diego, CA, USA), CD49f (clone GOH3; Biolegend, San Diego, CA, USA), CD66e (Serotec, Oxford, UK), CD90 (clone SE10; eBioescience), CD133 (Miltenyi Biotec), CD146 (BD Pharmingen), E-Cadherin (clone 67A4; Biolegend), EGFR (clone AY13; Biolegend), EpCAM (clone 9C4; Biolegend), human epidermal growth factor receptor 2/ HER2 (clone 24D2; Biolegend), and N-Cadherin (clone 8C11; BD Biosciences). Isotype matched antibodies were used as controls. Cells were analyzed employing a FACSCantoII (BD Biosciences) flow cytometer. Differentially expressed antigens were subsequently tested in the CD45^neg^ fraction (CTC enrichment; see later) of healthy donors to exclude those able to bind circulating cells in controls.

### Immunomagnetic enrichment and sample staining procedures

Blood samples (7.5 ml) were first subjected to red blood cell lysis, employing isotonic ammonium-chloride buffer, and then incubated for 20 minutes with CD45 MicroBeads (Miltenyi Biotec). Subsequently, samples were immunomagnetically depleted of the CD45^pos^ leukocyte fraction using LD separation columns in a MACS MIDI separator (Miltenyi Biotec), according to the manufacturer’s instructions. To evaluate the efficiency and the yield of the depletion strategy, both CD45^neg^ and CD45^pos^ fractions were labeled with CD45 and analyzed by fluorescence-activated cell sorting (FACS). CD45 depleted samples were subsequently stained with a cocktail of antibodies recognizing: epithelial markers (i.e., EpCAM, E-Cad) labeled by FITC, mesenchymal markers (i.e., CD44, CD146, and N-Cadherin) labeled by PE, and the pan-leukocyte CD45 marker labeled by APC.

### Spiked samples

For mimicking the in vivo presence of different subsets of CTC in the peripheral blood of MBC patients, known numbers of MCF-7 and MDA-MB231 cells, prelabeled with Hoechst 33342, were spiked into 7.5 ml of blood samples (*n =* 27) obtained from healthy donors. After erythrocyte lysis and immunomagnetic depletion of CD45^pos^ cells, the enriched samples were stained and analyzed by flow cytometry, as already described, to compute the yield of recovery, sensitivity, and specificity of the entire procedure.

### Immunofluorescence analysis of intracellular markers

For immunofluorescence analysis of intracellular markers, cells were fixed in 4% buffered paraformaldehyde, and permeabilized with 0.1% Triton X-100. Estrogen receptor (ER), vimentin, and cytokeratins were detected using the following monoclonal antibodies: anti-ERα (Clone SP1; ACZON, Monte San Pietro, BO, Italy), anti-vimentin (Clone V9; DAKO, Glostrup, Denmark), and anti-cytokeratins (8, 18, 19; BIO GENEX, San Ramon, CA, USA). Alexa Fluor 555 conjugated secondary antibodies (Invitrogen, Carlsbad, CA, USA) were used. Cells were analyzed by Leica DMI6000 B (Leica Microsystems, Wetzlar, Germany) utilizing a 40× oil immersion objective (numerical aperture: 1.25).

### CTC detection and sorting by the DEPArray

Blood samples, depleted of CD45^pos^ cells, were stained with Hoechst 33342 and the afore-described antibody cocktail. Stained cells were resuspended in 14 μl of RPMI1640 supplemented with 10% fetal bovine serum and 1% antibiotics (all from Invitrogen).

Cell sorting experiments were performed by DEPArray (Silicon Biosystems) as described in the manufacturer’s instructions. Briefly, DEPArray cartridges (A300K) were manually loaded with 14 μl of sample and 830 μl of culture medium. After loading the cartridge into the DEPArray system, the sample was injected by the system into a microchamber where the cells were exposed to an electric field consisting of 16,000 electrical cages in which individual cells are trapped. Image frames for each of the four fluorescent filters (FITC, PE, APC, and 4′,6-diamidino-2-phenylindole (DAPI)/Hoechst) and bright-field images were captured. Cell detection was based on a DAPI/Hoechst fluorescence threshold. For each cell, a unique ID was assigned. Captured images were processed and presented by the CellBrowser software that enables selection of cells of interest by the operator. Nucleated cells negative for CD45 were chosen, independently from the expression of epithelial and/or mesenchymal markers, and moved to a parking area in the cartridge. Individual cells were then subsequently moved to a recovery area where a last visual confirmation of cell presence could be performed. Typically, the procedure required about 2.5 hours for first recovery, with imaging of four channels including bright-field, and about 15 minutes for the recovery of each cell.

After DEPArray analysis, single tumor cells were recovered alive and then subjected to transcriptional analysis of target genes by multiplex reverse transcriptase PCR (RT-PCR).

### mRNA isolation and quantitative PCR analysis from single cells

After a single cell lysis step, performed according to the Ampli1 Whole Genome Amplification Kit (Silicon Biosystems) manufacturer’s instructions, the mRNA was isolated from the sample and reverse-transcribed into cDNA (Superscript; Invitrogen). All of the reagents required for quantitative RT-PCR were combined in a master mix (Universal SYBR Green Master Mix; Roche, Basel, Switzerland), including primers (Additional file 1: Table S1), probes, and enzyme (according to the manufacturer’s instructions). Then 14 μl aliquots of this solution and 1 μl of single-cell cDNA were dispensed in a 96-well plate. Real-time PCR and analyses were performed, employing a LightCycler 480 (Roche) instrument. For each amplification reaction, the melting temperature (Tm) and Cp values were computed.

### Statistical analysis

Patients’ characteristics have been summarized by means of descriptive analysis. Categorical variables were described by frequency distribution, whereas continuous variables were reported as median and interquartile range. Age, performance status, and number of lines were dichotomized, according to clinical interest, using respectively 70 years, ECOG PS 1, and the median of lines received (*n* = 2) as the threshold.

CTC subpopulation distributions were tested for normality by Shapiro–Wilk test and their association with clinicopathological features was explored by Wilcoxon rank-sum test or Kruskal–Wallis test, as appropriate. Owing to the exploratory purpose of the study, no corrections for multiple comparisons were applied. The prognostic impact of CTC count was investigated by univariate Cox regression models with 95 % confidence interval both in terms of overall survival (OS; calculated from both the stage IV diagnosis and the initial CTC assessment) and progression-free survival (PFS; calculated from CTC assessment to the first evidence of disease progression or death). The proportional hazard assumption was tested through the Schoenfeld residuals test.

Differences between CTC distribution quartiles were described by Kaplan–Meier estimator plot and tested by the log-rank test. *p* <0.05 was considered significant. Statistical analysis was conducted using StataCorp 2013 Stata Statistical Software: Release 13 (College Station, Texas, USA).

## Results

### Patient recruitment and baseline characteristics

This study enrolled 56 MBC patients treated at the University Hospital of Udine between March 2013 and May 2015, regardless of the line of treatment. CTC assessment was performed before the beginning of a new therapeutic line. Among them, 47 patients were eligible for CTC analysis. Table 1 summarizes patients’ clinicopathological characteristics and treatment history. Median age was 62 years (range 36–82). The most common histotype was ductal (84 %), while the most frequent subtype was luminal-like (54 %). MBC was diagnosed as de novo disease in 15 patients. The median number of previous therapeutic lines was 1 (range 0–10). At the time of recruitment, 66 % of patients presented at least one visceral site of metastatic spread, while 10 % of patients were affected by bone-only disease. At the database lock, disease progression was observed in 29 cases and death in 18 cases. Median follow-up was 33 months, median OS from CTC assessment was 21.7 months, while median PFS was 8.12 months. The estimated 1-year and 2-year OS rates were 70 % and 46 %, respectively, while the estimated 1-year and 2-year PFS rates were 37 % and 19 % respectively.

**Table 1:** Demographic and clinicopathological features of the 47 analyzed metastatic breast cancer patients

Age	
Median (range)	62 (36–82)
< 70 years	36 (77 %)
≥ 70 years	11 (23 %)
ECOG PS, *n* (%)	
0	29 (62 %)
1	14 (29 %)
2	4 (9 %)
Immunophenotype, *n* (%)	
Luminal-like	25 (54 %)
HER2-positive	11 (23 %)
Triple negative	11 (23 %)
Histotype, *n* (%)	
Ductal	38 (84 %)
Lobular	7 (16 %)
Metastatic sites,^a^ *n* (%)	
Bone	29 (62 %)
Liver	22 (47 %)
Lung	16 (34 %)
CNS	3 (6 %)
Number of metastatic sites, *n* (%)	
1	16 (34 %)
≥ 2	31 (66 %)
Number of previous lines, *n* (%)	
0	15 (32 %)
1	9 (19 %)
≥ 2	23 (49 %)
Type of treatments received, *n* (%)	
Chemotherapy	33 (70 %)
Endocrine therapy	6 (13 %)
Chemotherapy and endocrine therapy	5 (11 %)
Palliative care	2 (6 %)
Anti-HER2 targeted therapy	12 (26 %)

^a^Patients may have more than one site involved

*ECOG PS* Eastern Cooperative Oncology Group Performance Status, *CNS* central nervous system, *HER2* human epidermal growth factor receptor 2

### Identification of epithelial and mesenchymal CTC by means of surface antigen expression

**Fig. 1:**
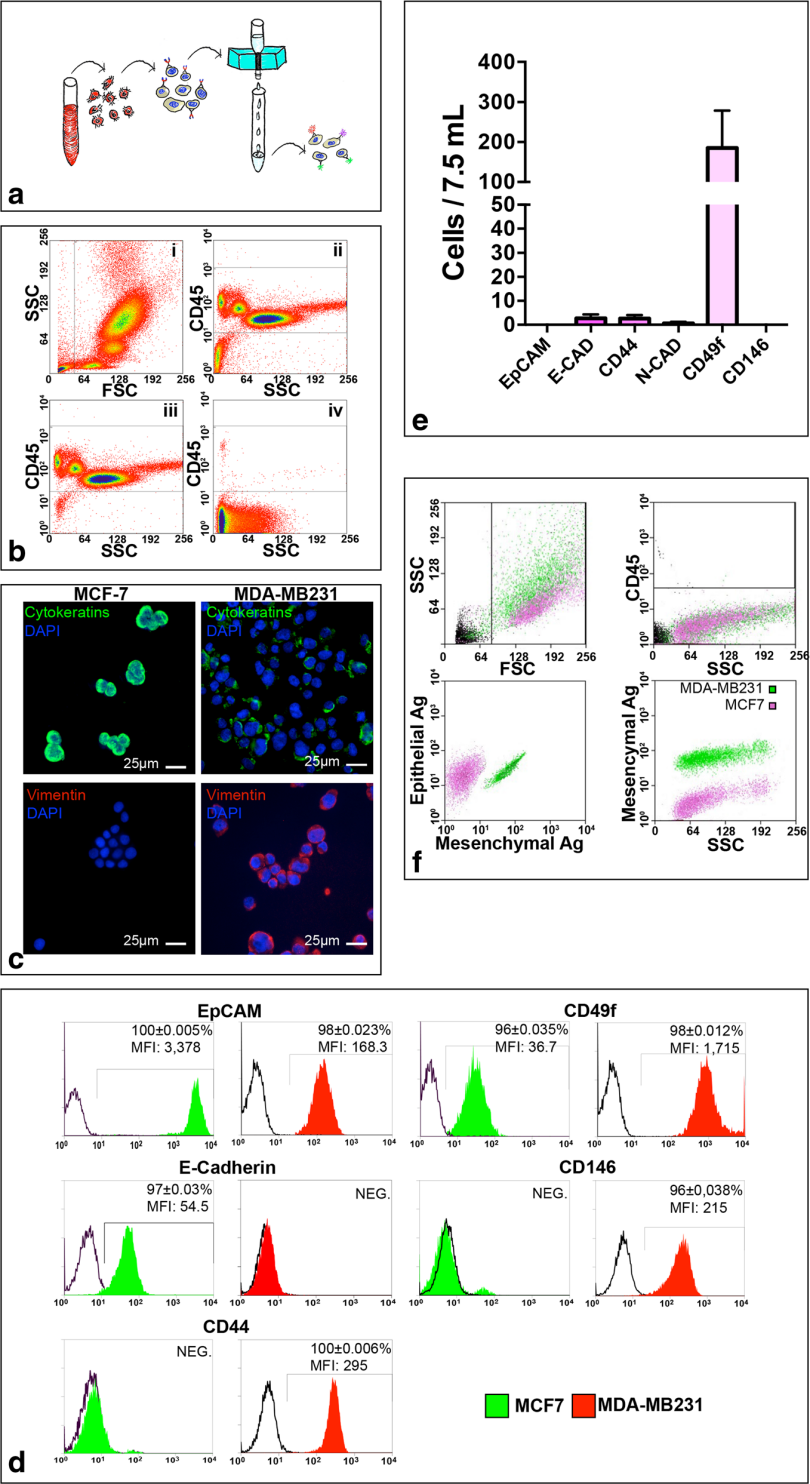
CTC enrichment strategy. **a** Scheme showing the enrichment strategy. **b** Dot plots showing physical parameters (*i*) and CD45 expression of cells before (*ii*) and after depletion (*iii*), (*iv*). CD45-positive (*iii*) and CD45-negative (*iv*) fractions are shown. **c** Immunofluorescence analysis of the epithelial-like (MCF7, *left panels*) and mesenchymal-like (MDA-MB231, *right panels*) breast cancer cell lines showing cytokeratin (*green fluorescence*) and vimentin (*red fluorescence*) expression. Nuclei are labeled by DAPI (*blue fluorescence*). **d** FACS analysis showing, in histograms, the differential expression of epithelial and mesenchymal surface antigens by MCF-7 (*green histograms*) and MDA-MB231 (*red histograms*) cell lines. *White histograms* show negative controls. **e** Histograms showing the estimated number of CD45-negative peripheral blood mononuclear cells, isolated from 7.5 ml of healthy blood female donors (*n* = 10), expressing either epithelial or mesenchymal antigens. **f** FACS plot showing the ability of the enrichment strategy to identify MCF7 and MDA-MB231 cells spiked into healthy donor peripheral blood samples. *DAPI* 4',6-diamidino-2-phenylindole, *EpCAM* epithelial cell adhesion molecule. (Color figure online), *FSC* forward scatter, *SSC* side scatter, *MFI* mean fluorescence intensity

A possible limitation of the strategies currently used for the enumeration of CTC from blood samples is the heterogeneous expression of EpCAM on tumor cells, especially on those undergoing EMT [20]. In order to circumvent this issue, we decided to adopt a CTC enrichment strategy based on red blood cell lysis followed by the immunomagnetic depletion of leukocytes from blood samples (i.e., a negative selection; Fig. 1a). The efficiency of this procedure was 99.98 ± 0.012 % (*n* = 8; Fig. 1b) and an average of 2656 ± 2531 cells/7.5 ml of processed blood (*n* = 6) could be recovered.

In order to prospectively identify both epithelial-like and mesenchymal-like CTC, we evaluated the surface antigen immunophenotype of two breast cancer lines considered to be prototypical epithelial-like (MCF7) and mesenchymal-like (MDA-MB231) tumor cells [21, 22]. MCF-7 and MDA-MB231 cells differed in the expression of EpCAM, E-Cadherin, CD44, CD49f, and CD146, the first two being upregulated in epithelial-like cells but the latter three in mesenchymal-like cells (Fig. 1c, d). Since our objective was to identify an antibody cocktail able to recognize tumor cells with high specificity, we employed flow cytometry to evaluate the expression of the candidate markers in the CD45-negative fraction of 10 healthy donors. In this way we could exclude those antigens that were commonly present in circulating cells. In fact, while CD49f was frequently observed in donor blood cells, E-Cadherin, EpCAM, CD146, and CD44 were almost undetectable in the CD45-negative fraction of female donors (Fig. 1e). Therefore, we tested, in spiked donor samples subjected to negative selection, an antibody cocktail recognizing epithelial (i.e., FITC-labeled anti-EpCAM, and anti-E-Cad), mesenchymal (i.e. PE-labeled anti-CD44, and anti-CD146), and leukocyte (i.e., APC-labeled anti-CD45) markers. This strategy was able to identify, in spiked samples (*n* = 27), Hoechst-labeled breast cancer cells with a sensitivity of 99 ± 1.4 %, a specificity of 99 ± 0.7 %, and a positive predictive value of 95 ± 5.7 %. Values were similar for epithelial-like breast tumor cells (99.7 ± 0.4 %, 98.4 ± 2 %, 90 ± 2.6 %) and mesenchymal-like breast tumor cells (98 ± 1.7 %, 99.9 ± 0.1 %, 99.9 ± 0.1 %) (Fig. 1f). To further confirm the specificity of the cocktail, this latter was tested in the CD45-negative fraction of 18 female donors; no epithelial cells were detected, while cells expressing only mesenchymal markers were documented, at low number, in three samples (Additional file 2: Table S2A).

### Enumeration and sorting of viable single CTC by DEPArray technology

**Fig. 2:**
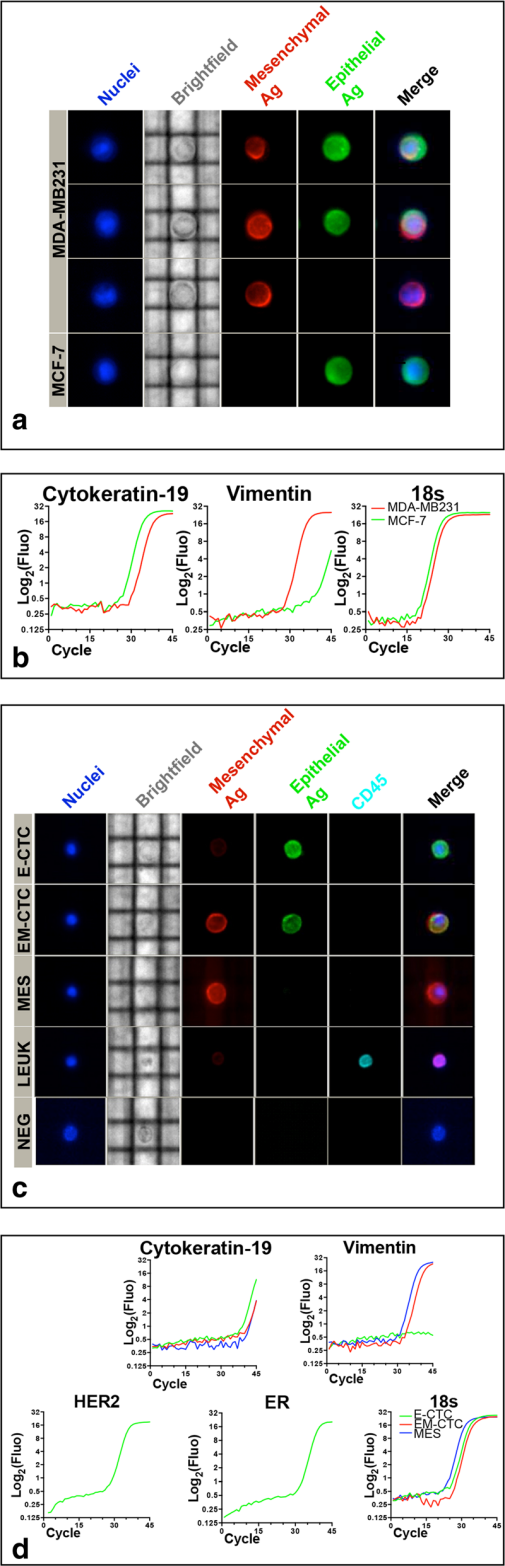
Enumeration and sorting of viable single CTC by DEPArray technology. **a** Image gallery of MDA-MB231 and MCF-7 cells, analyzed from a spiked sample, showing, from top to bottom, two cells coexpressing mesenchymal (*red fluorescence*) and epithelial (*green fluorescence*) markers, one cell expressing only mesenchymal markers, and one cell expressing only epithelial markers, respectively. Images of tumor cell morphology are shown in the bright-field channel (*grey*), while cell nuclei are stained by Hoechst (*blue*). **b** Real-time PCR showing cytokeratin-19 and vimentin expression in sorted, single MCF-7 (*green line*) and MDA-MB231 (*red line*) cells. **c** Representative images of the analysis of a blood sample from a MBC patient enriched employing the strategy described in Fig. 1a. The CD45-depleted fraction was labeled with antibodies recognizing epithelial (*green fluorescence*) and mesenchymal (*red fluorescence*) markers, as well as the common leukocyte antigen CD45 (*cyan fluorescence*). The image bar displays, from top to bottom, one cell expressing only epithelial markers (*E CTC*), one cell coexpressing mesenchymal and epithelial markers (*EM CTC*), one cell expressing only mesenchymal markers (*MES*), one CD45-positive leukocyte (*LEUK*), and one cell negative for all of the tested markers (*NEG*), respectively. Images of tumor cell morphology are shown in the bright-field channel (*grey*), while cell nuclei are stained by Hoechst (*blue*). **d** Real-time PCR showing cytokeratin-19, vimentin, HER2, and ER expression in sorted, single E-CTC (*green line*), EM-CTC (*red line*), and MES (*blue line*) cells (Color figure online)

We took advantage of the DEPArray system to identify and sort single, viable CTC, based on a multiparametric fluorescence analysis. Specifically, spiked samples containing epithelial-like and/or mesenchymal-like breast tumor cells were stained with Hoechst 33342 and the afore-described antibody cocktail. This approach allowed us to identify and sort single breast tumor cells as viable nucleated cells, negative for CD45 and expressing epithelial and/or mesenchymal markers (Fig. 2a). Sorted cells were suitable for RNA isolation followed by real-time PCR analysis of transcripts typical of epithelial and EMT cells (Fig. 2b).

Once the isolation protocol was optimized, we analyzed blood samples obtained from enrolled patients. In this case, to the afore-described antibody cocktail we added N-Cadherin, a marker of EMT [23] not expressed on human CD45^neg^ cells (Fig. 1e). On the basis of the DEPArray analysis, CD45-negative cells were classified as epithelial CTC (expressing only epithelial markers (E CTC)), CTC in EMT (coexpressing epithelial and mesenchymal markers (EM CTC)), putative mesenchymal cells (expressing only mesenchymal markers (MES)), and negative cells (not expressing the tested markers (NEG)). Raw data are presented in Additional file 3: Table S3. All of these cell types could be detected and sorted (Fig. 2c and Table 2). Specifically, the median number of 96 CD45^neg^ cells per 7.5 ml of peripheral blood was counted. Of these, more than 2/3 were expressing only mesenchymal markers, 4 % were negative for all of the tested antibodies, and a median fraction of 16 % was positive for the analyzed epithelial markers. Of these latter cells, positive to the epithelial cocktail and therefore considered as bona fide CTC, 22 % coexpressed mesenchymal markers. When the same protocol was applied to blood samples obtained from three female donors, no E CTC, EM CTC, and MES cells were detected (Additional file 2: Table S2B), confirming the absence of bona fide CTC in healthy donors. Conversely, NEG cells were documented in all samples, although at a low number (Additional file 2: Table S2B).

**Table 2:** CD45^neg^ subpopulations at baseline

Cell class	Immunophenotype	Median (25^th^; 75^th^)
E CTC^a^	E^+^M^−^	8 (3; 24)
EM CTC^a^	E^+^M^+^	3 (0; 9)
MES^a^	E^−^M^+^	45 (15; 95)
NEG^a^	E^−^M^−^	4 (0; 14)
Derived parameters	Function	Median (25^th^; 75^th^)
E^TOT^ CTC^a^	EM CTC + E CTC	10 (4; 33)
CD45^nega^	ETOT CTC + MES + NEG	96 (42; 145)
EM CTC fraction over CD45^neg^ ^b^	EM CTC/CD45^neg^ × 100	2 (0; 8)
ETOT CTC fraction over CD45^neg b^	ETOT CTC/CD45^neg^ × 100	16 (5; 30)
MES fraction over CD45^neg b^	MES/CD45^neg^ × 100	79 (40; 88)
NEG fraction over CD45^neg b^	NEG/CD45^neg^ × 100	4 (0; 15)

Summary of the definition of the cell subpopulations counted by DEPArray, the parameters computed from these absolute cell numbers, and the descriptive statistics, expressed as median and interquartile range, of cell classes and derived parameters (*n* = 47)

^a^Results expressed as absolute number of cells for 7.5 ml of peripheral blood

^b^Results expressed as percentage

*E* reactivity to the epithelial antibody cocktail, *E CTC* subset of CD45^neg^ expressing only epithelial markers, *EM CTC* subset of CD45^neg^ coexpressing mesenchymal and epithelial markers, *ETOT CTC* CD45^neg^ cells expressing epithelial markers independently from the expression of mesenchymal markers, *M* reactivity to the mesenchymal antibody cocktail, *MES* subset of CD45^neg^ expressing mesenchymal markers only, *NEG* subset of CD45^neg^ negative to the antibody cocktail

Downstream mRNA analysis supported the classification based on the surface immmunophenotype. Specifically, single sorted epithelial cells did not express vimentin, while cells in EMT expressed both vimentin and cytokeratin 19, and mesenchymal cells expressed vimentin only. Additionally, in patient-derived E CTC, the expression of HER2 and ER transcripts could also be demonstrated (Fig. 2d).

Altogether these findings indicate that the DEPArray-based CTC detection protocol we optimized could identify CTC subsets in clinical samples obtained from MBC patients. Importantly, the collection of viable, single CTC followed by gene expression analysis corroborates our classification based on cell surface immunophenotype.

### Association between CTC subpopulations and clinicopathological features

**Fig. 3:**
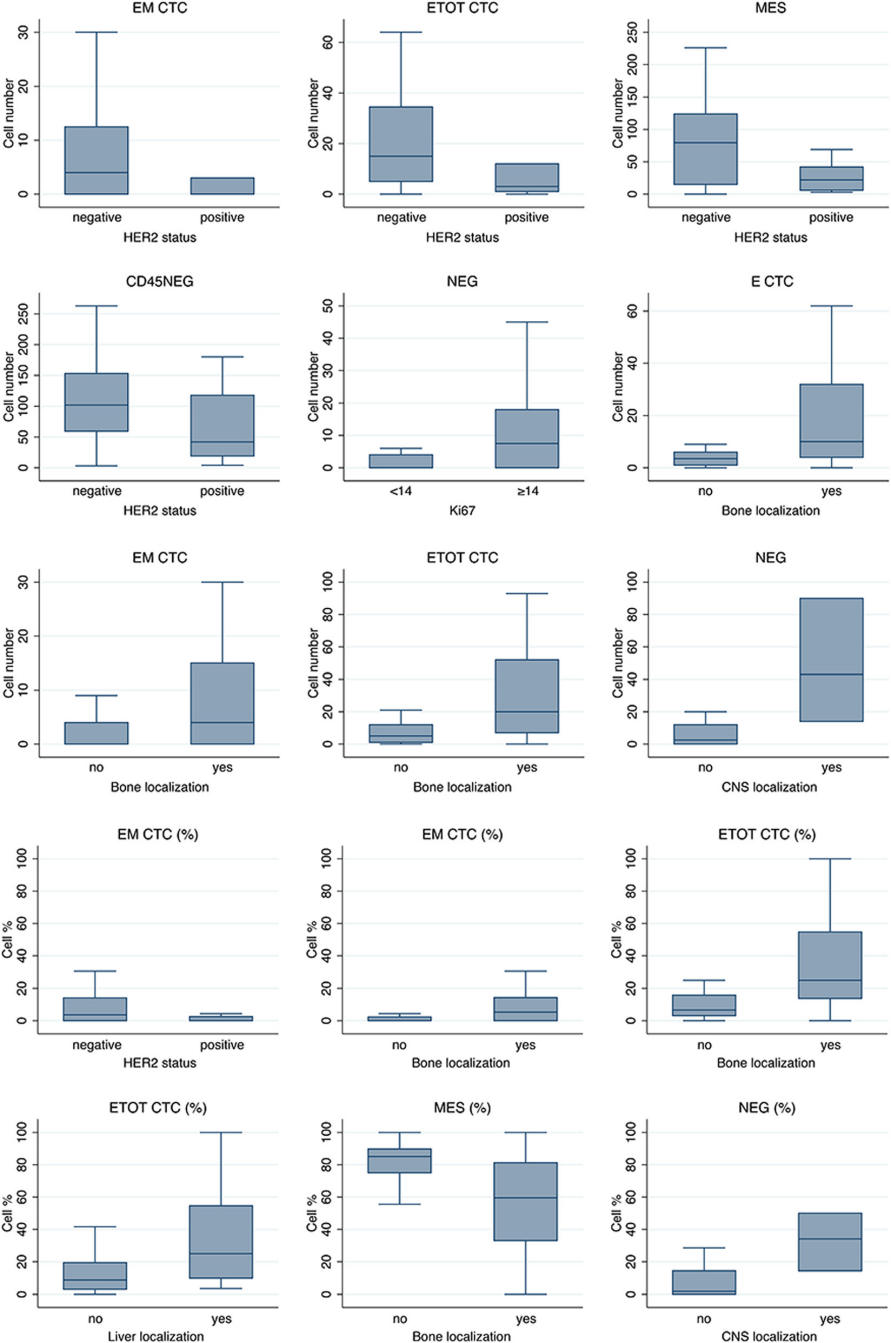
Association between CTC subpopulations and clinicopathological characteristics. Box and whiskers plots showing median, interquartile ranges, 5th and 95th percentile of the absolute number or frequency of different classes of cells (E, E/M, MES, NEG) significantly associated with specific clinicopathological features. *E CTC* cells expressing only epithelial markers, *EM CTC* cells coexpressing mesenchymal and epithelial markers, *ETOT CTC* CD45^neg^ cells expressing epithelial markers, independently from the expression of mesenchymal markers, *MES* cells expressing only mesenchymal markers, *NEG* cells negative for all of the tested markers, *CNS* central nervous system, *HER2* human epidermal growth factor receptor 2

Next, we assessed the association between the clinicopathological characteristics of MBC patients and the different subtypes of circulating cells that were identified, as already described, by DEPArray (Table 3, Fig. 3).

**Table 3:** Association between circulating CD45^neg^ subpopulations and clinicopathological features of MBC patients

		Absolute number						Fraction over CD45^neg^ (%)			
		E CTC	EM CTC	ETOT CTC	MES	NEG	CD45^neg^	EM CTC	ETOT CTC	MES	NEG
Tumor characteristics											
HER2	Positive (*N* = 11)	3 (0; 9)	**0** ** (0; 3)**	**3** ** (1; 12)**	**22** ** (6; 42)**	0 (0; 14)	**42** ** (19; 118)**	**0** ** (0; 3)**	16 (4; 25)	81 (33; 97)	0 (0; 38)
	Negative (*N* = 36)	8 (4; 27)	**4** ** (0; 13)**	**15** ** (5; 35)**	**80** ** (15; 124)**	4 (0; 13)	**102** ** (60; 153)**	**4** ** (0; 14)**	17 (6; 39)	74 (40; 88)	4 (0; 15)
	p*	0.12	**0.029**	**0.037**	**0.022**	0.84	**0.046**	**0.035**	0.37	0.70	0.94
Profile	Luminal (*N* = 25)	8 (4; 32)	**4** ** (1; 15)**	16 (6; 53)	72 (14; 103)	0 (0; 9)	97 (59; 197)	4 (0; 14)	19 (6; 55)	70 (39; 90)	0 (0; 6)
	HER2-positive (*N* = 11)	3 (0; 9)	**0** ** (0; 3)**	3 (1; 12)	22 (6; 42)	0 (0; 14)	42 (19; 118)	0 (0; 3)	16 (4; 25)	81 (33; 96)	0 (0; 38)
	TNBC (*N* = 11)	9 (2; 24)	**3** ** (0; 9)**	15 (2; 30)	84 (29; 125)	9 (5; 18)	124 (60; 150)	2 (0; 8)	16 (3; 21)	79 (60; 86)	15 (4; 16)
	p**	0.30	**0.044**	0.087	0.052	0.092	0.12	0.064	0.35	0.93	0.056
Ki-67	<14 % (*N* = 10)	7 (4; 35)	7 (0; 18)	18 (6; 53)	42 (6; 103)	**0** ** (0; 4)**	76 (41; 197)	4 (0; 31)	19 (8; 63)	63 (37; 90)	0 (0; 5)
	≥14 % (*N* = 34)	8 (2; 16)	2 (0; 6)	10 (3; 30)	55 (20; 92)	**8** ** (0; 18)**	99 (42; 138)	2 (0; 7)	16 (5; 25)	77 (41; 86)	8 (0; 16)
	p*	0.45	0.15	0.37	0.64	**0.039**	0.74	0.33	0.39	0.90	0.087
Metastatic site											
Bone	Yes (*N* = 29)	**10** ** (4; 32)**	**4** ** (0; 15)**	**20** ** (7; 52)**	45 (14; 84)	2 (0; 12)	95 (48; 126)	**5** ** (0; 14)**	**25** ** (14; 55)**	**60** ** (33; 81)**	2 (0; 14)
	No (*N* = 29)	**4** ** (1; 6)**	**0** ** (0; 4)**	**5** ** (1; 12)**	55 (20; 129)	6 (0; 18)	89 (23; 150)	**0** ** (0; 2)**	**7** ** (3; 16**	**85** ** (75; 90)**	6 (0; 16)
	p*	**0.0072**	**0.024**	**0.0058**	0.34	0.43	0.97	**0.0096**	**0.0006**	**0.012**	0.48
Liver	Yes (*N* = 22)	9 (4; 27)	3 (0; 9)	15 (4; 45)	42 (15; 75)	6 (0; 14)	90 (41; 126)	3 (0; 14)	**25** ** (10; 55)**	63 (33; 84)	4 (0; 15)
	No (*N* = 25)	6 (1; 10)	3 (0; 9)	10 (2; 21)	84 (20; 123)	3 (0; 12)	103 (42; 156)	2 (0; 6)	**9** ** (3; 19)**	81 (58; 89)	2 (0; 15)
	p*	0.28	0.99	0.27	0.28	0.87	0.47	0.48	**0.011**	0.067	0.86
Lung	Yes (*N* = 16)	9 (4; 20)	1 (0; 6)	10 (6; 30)	57 (23; 126)	6 (0; 13)	87 (42; 153)	3 (0; 14)	12 (4; 30)	79 (49; 87)	5 (0; 16)
	No (*N* = 31)	6 (2; 27)	3 (0; 15)	10 (3; 45)	45 (10; 92)	3 (0; 14)	95 (23; 135)	0 (0; 6)	17 (6; 33)	75 (33; 89)	2 (0; 15)
	p*	0.69	0.23	0.86	0.26	0.71	0.72	0.16	0.36	0.50	0.74
CNS	Yes (*N* = 3)	24 (8; 47)	6 (0; 21)	45 (8; 53)	45 (30; 75)	**43** ** (14; 90)**	126 (97; 180)	6 (0; 12)	25 (6; 55)	31 (25; 60)	**34** ** (14; 50)**
	No (*N* = 44)	6 (2; 20)	3 (0; 9)	10 (3; 32)	53 (15; 100)	**3** ** (0; 12)**	85 (37; 143)	2 (0; 10)	16 (5; 32)	79 (40; 89)	**2** ** (0; 15)**
	p*	0.16	0.56	0.26	1.0000	**0.016**	0.26	0.75	0.51	0.082	**0.020**
Number of sites	1 (*N* = 16)	5 (2; 9)	1 (0; 10)	7 (2; 17)	55 (13; 93)	1 (0; 9)	82 (41; 122)	2 (0; 9)	9 (3; 21)	82 (47; 91)	1 (0; 14)
	≥ 2 (*N* = 31)	9 (3; 27)	3 (0; 9)	15 (4; 36)	45 (15; 123)	5 (0; 14)	97 (41; 156)	2 (0; 12)	19 (6; 36)	70 (37; 86)	5 (0; 16)
	p*	0.22	0.76	0.19	0.84	0.40	0.67	0.78	0.16	0.28	0.61

Results are expressed as median (25th, 75th percentile). Bold cells indicate significant (*p* <0.05) differences as detected by Wilcoxon rank-sum test (*) or Kruskal-Wallis test (**), as appropriate

*E CTC* subset of CD45^neg^ expressing epithelial markers only, *EM CTC* subset of CD45^neg^ coexpressing mesenchymal and epithelial markers, *ETOT CTC* CD45^neg^ cells expressing epithelial markers independently from the expression of mesenchymal markers, *MBC* metastatic breast cancer, *MES* subset of CD45^neg^ expressing mesenchymal markers only, *NEG* subset of CD45^neg^ negative to the antibody cocktail, *CNS* central nervous system, *HER2* human epidermal growth factor receptor 2

Patients affected by HER2-positive disease, with respect to those affected by HER2-negative disease, had a significantly lower number of MES cells (*p* = 0.022), EM CTC (both in absolute number and in percentage, *p* = 0.029 and *p* = 0.035, respectively), ETOT CTC (*p* = 0.037), and CD45^neg^ (*p* = 0.046). Intriguingly, patients affected by triple-negative disease showed, with respect to those affected by other profiles, a significantly higher proportion and absolute number of NEG cells (*p* = 0.024 and *p* = 0.033, respectively). The presence of bone metastases was significantly associated with an increase in the absolute number of E CTC (*p* = 0.0074) and EM CTC (*p* = 0.024) and with both the absolute number (*p* = 0.0058) and the percentage (*p* = 0.00060) of CTC expressing epithelial markers (irrespective of the expression of mesenchymal markers (ETOT CTC)). Additionally, bone metastases were associated with a lower percentage of MES cells (*p* = 0.012), while patients with liver localizations were characterized by a higher proportion of E CTC (*p* = 0.011). Of note, patients with central nervous system involvement were characterized by a higher number of circulating NEG cells, both when considered as absolute numbers (*p* = 0.016) and as a percentage (*p* = 0.020). A higher absolute number of negative cells was also associated with highly proliferating primary breast tumors (Ki67 ≥ 14%; *p* = 0.039).

Interestingly, even if marginally significant, a lower percentage of MES cells was observed among patients with liver localizations and CNS involvement.

The number of metastatic sites did not seem to influence the number or proportion of different CTC populations.

Altogether these results indicate that important clinical features of MBC patients are associated with distinct subpopulations of circulating cells. Therefore, the in-depth characterization of CD45^neg^ cells may be endowed with a prognostic or predictive significance.

### Exploration of the prognostic role of CTC

Lastly, we evaluated whether the enumeration of CD45^neg^ subpopulations by the DEPArray system could predict outcome.

Univariate Cox regression analysis showed a prognostic role of both ETOT CTC and EM CTC, as a continuous percentage variable, in terms of both OS from stage IV diagnosis (*p* = 0.015 and *p* = 0.022, respectively) and from CTC assessment (*p* = 0.013 and *p* = 0.0016, respectively). However, the proportion of EM CTC was the only parameter that resulted to be significantly associated with PFS (*p* = 0.016). Intriguingly, the proportion of MES/CD45^neg^ had a favorable impact in terms of OS (from stage IV diagnosis *p* = 0.037). The full set of variables investigated in the univariate analysis is reported in Table 4.

**Table 4:** Univariate analysis of demographic data, clinicopathological data, and circulating CD45^neg^ subpopulations with PFS and OS, computed from both the diagnosis of metastasis and the CTC assessment

Variable		OS (from stage IV diagnosis)			OS (from CTC assessment)			PFS		
		HR	95 % CI	*p*	HR	95 % CI	*p*	HR	95 % CI	*p*
ECOG PS	2-3 vs. 0-1	**5.288**	**1.392–20.096**	**0.015**	**11.294**	**2.467–51.702**	**0.0018**	2.558	0.730–8.963	0.14
BC profile	HER2-positive vs. luminal	0.338	0.075–1.525	0.16	0.274	0.061–1.235	0.092	0.616	0.237–1.599	0.32
	TNBC vs. luminal	1.803	0.564–5.771	0.32	0.972	0.312–3.029	0.96	1.212	0.495–2.963	0.67
Lines received	> 2 vs. < 2	**3.452**	**1.138–10.475**	**0.029**	0.622	0.245–1.581	0.32	0.557	0.265–1.172	0.12
Age	≥ 70 vs. < 70	0.593	0.19–1.809	0.34	0.652	0.213–1.997	0.45	1.031	0.459–2.314	0.94
E CTC (*n*)		1.003	0.982–1.024	0.79	1.001	0.980–1.023	0.92	1.004	0.989–1.020	0.6
EM CTC (*n*)		1.018	0.994–1.043	0.13	1.023	0.998–1.049	0.08	1.020	0.998–1.043	0.081
ETOT CTC (*n*)		1.006	0.993–1.019	0.35	1.006	0.993–1.019	0.38	1.007	0.996–1.018	0.24
MES (*n*)		0.998	0.991–1.005	0.56	0.998	0.991–1.005	0.56	0.998	0.992–1.003	0.41
NEG (*n*)		0.998	0.981–1.015	0.79	0.992	0.975–1.010	0.41	0.997	0.986–1.008	0.58
CD45^neg^ (*n*)		0.999	0.994–1.004	0.79	0.999	0.993–1.004	0.61	0.999	0.995–1.003	0.56
EM CTC/CD45^neg^ (%)		**1.022**	**1.003–1.042**	**0.022**	**1.035**	**1.013–1.057**	**0.0016**	**1.021**	**1.004–1.039**	**0.016**
E TOT/CD45^neg^ (%)		**1.019**	**1.004–1.034**	**0.015**	**1.019**	**1.004–1.034**	**0.013**	1.010	0.997–1.023	0.12
MES/CD45^neg^ (%)		**0.984**	**0.968–0.999**	**0.037**	0.988	0.973–1.002	0.1	0.993	0.981–1.005	0.26
NEG/CD45^neg^ (%)		0.999	0.972–1.027	0.95	0.992	0.966–1.018	0.54	0.998	0.980–1.017	0.86

Bold cells indicate significant (*p* <0.05) association as detected by univariate Cox regression model

*CI* confidence interval, *CTC* circulating tumor cells, *ECOG PS* Eastern Cooperative Oncology Group Performance Status, *E CTC* subset of CD45^neg^ expressing epithelial markers only, *EM CTC* subset of CD45^neg^ coexpressing mesenchymal and epithelial markers, *ETOT CTC* CD45^neg^ cells expressing epithelial markers independently from the expression of mesenchymal markers, *HR* hazard ratio, *MES* subset of CD45^neg^ expressing mesenchymal markers only, *NEG* subset of CD45^neg^ negative to the antibody cocktail, *OS* overall survival, *PFS* progression-free survival, *BC* breast cancer, *HER2* human epidermal growth factor receptor 2, *TNBC* triple negative breast cancer

**Fig. 4:**
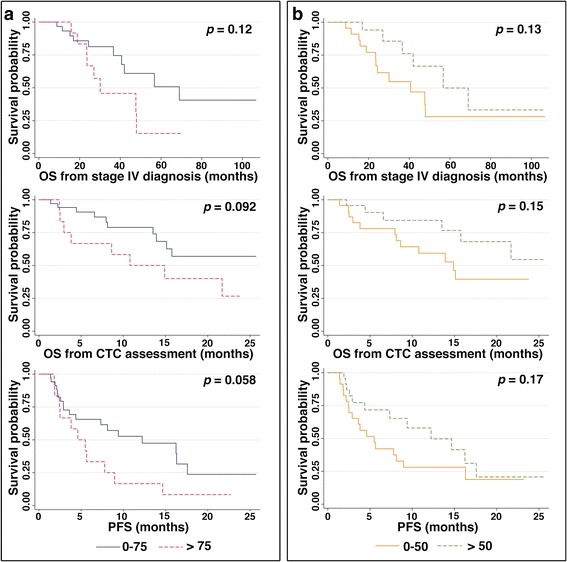
Survival curves. **a** Kaplan–Meier estimator plots in terms of OS from stage IV diagnosis (*upper panel*), OS from CTC assessment (*middle panel*), and PFS (*lower panel*) in patients with MBC for those with a fraction of EM CTC above the 75^th^ percentile (*dotted red line*) and for those with a fraction of EM CTC less than or equal to the 75^th^ percentile (*solid black line*) before initiation of a new line of therapy. **b** Kaplan–Meier estimates in terms of OS from stage IV diagnosis (*upper panel*), OS from CTC assessment (*middle panel*), and PFS (*lower panel*) in patients with MBC for those with a fraction of MES above the 50th percentile (*dotted green line*) and for those with a fraction of MES less than or equal to the 50th percentile (*solid orange line*) before initiation of a new line of therapy. *CTC* circulating tumor cells, *OS* overall survival, *PFS* progression-free survival. (Color figure online)

Furthermore, we assessed that stratifying the MBC population according to the percentage of EM CTC and MES could help in describing prognosis. The resulting Kaplan–Meier estimator plots are shown in Fig. 4.

Altogether these results indicate that the identification of the subpopulation of E CTC coexpressing mesenchymal markers may help in discriminating the subset of patients that are at high risk for disease progression.

## Discussion

Over the last 10 years, a general consensus has been reached on the role exerted by the quantitative analysis of CTC in the prognostic stratification of patients with MBC [1, 5, 8]. As a consequence, it has been suggested to employ CTC as a liquid biopsy to perform real-time monitoring of the metastatic status of a given patient [24], possibly offering an important parameter for precision medicine and personalized treatments [25].

The only FDA-approved instrument for the enumeration of CTC is, thus far, the CellSearch System (Veridex) [26]. Although this latter is robust and its ability to predict patients’ prognosis has been validated over the years [5, 8, 27], several potential limitations have been described. Specifically, the CellSearch System processes fixed blood samples and selects CTC from blood cells relying on the expression of EpCAM on their surface. However, several investigators [28, 29] have raised concerns regarding the homogeneous expression of this antigen on CTC. This issue is especially prominent in cells undergoing EMT, a process that downregulates typical epithelial markers (such as EpCAM, E-Cadherin, and keratins), upregulates mesenchymal markers (e.g. vimentin), and is characterized by reduced adhesive properties and increased invasiveness [20]. To overcome this major intrinsic limitation of the methodology, in recent years several investigators have tried to develop alternative assays that either target antigens alternative to EpCAM (e.g., CD146, CD49f) [29–31] or take advantage of different, antigen-independent, techniques (e.g., PCR-based assays, density gradient centrifugation, cell filtration) [1, 20]. However, the latter techniques, still at an experimental stage, are not approved for diagnostic purposes because they lack a clinical validation [14].

In this work, we have optimized an alternative enrichment strategy, taking into account that, as previously stated, the “perfect” CTC marker should be expressed on all CTC, but not on autochthonous blood cells [1]. Therefore, we first depleted leukocytes employing anti-CD45 immunomagnetic beads. Subsequently, we selected the antibody cocktail that showed the best performance in discriminating epithelial and mesenchymal surface antigens on CTC. For this purpose we screened 17 antigens to identify those that were differentially expressed by a luminal-like cell line (i.e., MCF7) and a triple negative-like cell line (i.e., MDA-MB231). Candidate antigens were further refined to exclude those that were expressed on circulating, CD45^neg^ cells, in female blood donors. This way we decided that, as opposed to a previous report [31], CD49f was not a useful marker in our hands. Employing this strategy, we were able to sort with high reproducibility breast cancer cells from spiked samples.

Another possible limitation of the CellSearch System is its inability to sort single, viable, and unfixed CTC to perform further downstream analyses. To circumvent this problem, we employed a dielectrophoresis-based system [32] (i.e., the DEPArray system) that has the ability to perform fluorescence microscopy analysis, mobilization, and collection of single viable cells from a pool of up ≈ 100,000 cells. In our hands, the labeling strategy that we optimized was a perfect match for the DEPArray system. In fact, the immunodepletion approach resulted in a number of recovered cells that was compatible with the loading capacity of the DEPArray chip (but avoids the loss of cells that do not express EpCAM), and the multiparametric analysis, based on an antibody cocktail recognizing both epithelial and mesenchymal antigens, allowed the identification of different cell subtypes. Last, our approach permitted us to isolate single, viable CTC [33] that could be further analyzed by quantitative RT-PCR. As a result, however, our method of analysis is slower than the method employed by the CellSearch System. In fact, with the workflow for sample preparation and analysis suggested in the manuscript, two patients per day can be processed. However, we can obtain, for each analyzed patient, an improved evaluation of the CTC heterogeneity, and molecular studies of single sorted CTC might add valuable prognostic and predictive information.  By systematically applying our analysis to 47 blood samples obtained from MBC patients, we observed four different cell subpopulations: CTC expressing only epithelial antigens, CTC coexpressing epithelial and mesenchymal antigens, cells expressing only mesenchymal antigens, and cells that do not express either epithelial or mesenchymal antigens. While the first two cell types are obviously CTC, less clear is the neoplastic nature of the latter two cell types. Ongoing single-cell genomic analysis experiments will clarify this issue (data not shown).

However, the more in-depth analysis of the CD45^neg^ fraction has allowed us to identify specific circulating cell subsets that are significantly associated with relevant clinical parameters. In fact, we showed that different breast tumor subtypes are associated with a distinct pattern of circulating CD45^neg^ cell subpopulations. Additionally, we observed that HER2-positive tumors were characterized by a trend to a reduced number of circulating MES and ETOT CTC. This finding is in line with data obtained by Cristofanilli’s group [34] and could possibly be related to the targeted therapy to which the MBC patient population is exposed. In fact, it has been shown that herceptin can downregulate the expression of chemokine (C-X-C motif) receptor 4 (CXCR4), which is required for HER2-enhanced invasion, migration, and metastasis [35]. Conversely, triple-negative tumors, with respect to the luminal ones, were associated with an increased absolute number and relative number of circulating cells not expressing either mesenchymal or epithelial markers. This evidence suggests that a fraction of CTC not recognized by the antibody cocktail that we employed may be comprised in this cell subset. Importantly, our approach allowed us to sort this cell population, which is now available for downstream molecular analyses. Ongoing DNA sequencing experiments will verify the presence of mutations shared with the tumor of origin, while future gene expression profiling experiments will help in selecting additional surface proteins that could be used to further refine our procedure.

Concerning the association between metastatic sites and the CD45^neg^ cell subsets, an increased number of NEG cells was significantly associated with the presence of brain secondary lesions. This result is in line with literature data showing that CTC with brain metastatic potential are indeed EpCAM-negative [4] and supports the importance of going beyond the pure enumeration of CTC expressing this antigen. Conversely, bone metastases were strongly associated with the absolute and relative abundance of CTC expressing epithelial antigens. This finding is consistent with the observed association of bone metastases with a CTC number ≥ 5 cells/7.5 ml of peripheral blood, as estimated by CellSearch [36].

Last, we assessed whether the identification of CTC in EMT was endowed with a prognostic significance. Specifically, the fraction of CD45^neg^ cells coexpressing epithelial and mesenchymal markers was associated with both computed patient PFS and OS, the latter both from the diagnosis of stage IV disease and from the CTC assessment. We decided to evaluate the overall survival from both enrollment into the study and from the diagnosis of metastasis, because, conceptually, these are the expression of two different views. The first considers the cancer as characterized by a site-dependent and time-related tumor heterogeneity that can be caused by both intrinsic (tumor-related) and extrinsic (therapy-related) factors [37], and CTC analyses have been considered a way to perform real-time monitoring of the metastatic disease by means of a minimally invasive technique (liquid biopsy) [20]. Upon the second view, CTC measurements could predict the intrinsic drug resistance of the metastatic disease. In our prospective observational study, the ability of CTC in EMT to predict the OS from the diagnosis of the metastatic disease suggests the existence of a subset of tumors whose prognosis is not significantly modified by currently available therapies. A similar conclusion has been reached by the clinical study SWOG S0500 [38], where early switching to an alternate cytotoxic therapy in patients with persistently increased CTC after 21 days of first-line chemotherapy was not effective in prolonging OS. Those authors suggested that it would be more profitable for this population of patients to be recruited into prospective trials of novel therapies and to take advantage of molecular analyses of metastasis, CTC, or circulating cell-free DNA to guide therapy [38].

It is important to underline that because of the relatively small sample size, the results from this observational study must be treated cautiously. Accordingly, validation of these findings in a larger independent cohort of patients is needed.

## Conclusions

In this work we optimized a novel strategy to enrich blood samples in CTC, independent from the expression of epithelial markers. Moreover, taking advantage of the DEPArray system, we have identified and sorted, based on multiparametric fluorescence analysis, single, viable epithelial-like CTC as well as CTC in EMT. Intriguingly, our CTC enrichment strategy allowed us to observe two additional circulating subsets in the CD45^neg^ fraction: one expressing only mesenchymal surface proteins, and the other negative for all of the tested markers. The neoplastic nature of these cell populations is still an open issue. Nonetheless, each of these CD45^neg^ cell subsets was associated, in a prospective study including 47 patients affected by MBC, with clinically relevant parameters, such as tumor subtype, Ki67 expressions and the site of metastasis. Most importantly, the fraction of CTC expressing EMT markers was an independent predictor of poor outcome.

Altogether these results indicate that the protocol that we optimized for the analysis and sorting of circulating CD45^neg^ cells in MBC patients could be of high clinical relevance and deserves validation in a larger independent cohort of patients.

## Additional files

Additional file 1: Table S1. Presenting primers pairs for real-time PCR. (DOCX 14 kb) Additional file 2: Table S2A. Presenting enumeration of different CD45^neg^ subgroups, as assessed by FACS, in blood samples obtained from healthy female donors, and **Table S2B.** presenting enumeration of different CD45^neg^ subgroups, as assessed by DEPArray, in blood samples obtained from healthy female donors. (DOCX 90 kb) Additional file 3: Table S3. Presenting the CTC count at baseline in each analyzed patient. (DOCX 18 kb)

## Abbreviations

APC: Allophycocyanin
CD45^neg^: circulating cells not expressing CD45
CTC: Circulating tumor cells
CXCR4: Chemokine (C-X-C motif) receptor 4
DAPI: 4′,6-Diamidino-2-phenylindole
E CTC: Subset of CD45^neg^ expressing epithelial markers only
ECOG PS: Eastern Cooperative Oncology Group Performance Status
EMT: Epithelial-to-mesenchymal transition
EpCAM: Epithelial cell adhesion molecule
ER: Estrogen receptor
ETOT CTC: CD45^neg^ cells expressing epithelial markers, independently from the expression of mesenchymal markers
FACS: Fluorescence-activated cell sorting
FDA: Food and Drug Administration
FITC: Fluorescein isothiocyanate
MBC: Metastatic breast cancer
MES: Subset of CD45^neg^ expressing mesenchymal markers only
NEG: Subset of CD45^neg^ negative to an antibody cocktail (including CD45, EpCam, E-Cadherin, N-Cadherin, CD44, and CD146)
OS: Overall survival
PE: Phycoerythrin
PFS: Progression-free survival
RECIST: Response Evaluation Criteria In Solid Tumors
RT-PCR: Reverse transcriptase-PCR
