# Supplementary material for: In patients with metastatic breast cancer the identification of circulating tumor cells in epithelial-to-mesenchymal transition is associated with a poor prognosis
Source: Breast Cancer Res. 2016 Mar 9;18:30. doi: 10.1186/s13058-016-0687-3 (PMC4784394; doi:10.1186/s13058-016-0687-3)
Supplement: Additional file 1: Table S1. — Presenting primers pairs for real-time PCR. (DOCX 14 kb) [file 13058_2016_687_MOESM1_ESM.docx]

| GENE | *primer forward* | *primer reverse* |
| --- | --- | --- |
| **KRT19** | TCAGCGGTATTGAAGCCCAG | GGTAGGTGGCAATCTCCTGC |
| **ERBB2** | GAACTCACCTACCTGCCCAC | GACCTGCCTCACTTGGTTGT |
| **ESR1** | TCTTGGACAGGAACCAGGG | TGATGTAGCCAGCAGCATGT |
| **VIM** | GGCTCGTCACCTTCGTGAAT | GCAGAGAAATCCTGCTCTCCT |
| **RNA 18S5** | GTAACCCGTTGAACCCCATT | CCATCCAATCGGTAGTAGCG |
