# Supplementary material for: In patients with metastatic breast cancer the identification of circulating tumor cells in epithelial-to-mesenchymal transition is associated with a poor prognosis
Source: Breast Cancer Res. 2016 Mar 9;18:30. doi: 10.1186/s13058-016-0687-3 (PMC4784394; doi:10.1186/s13058-016-0687-3)
Supplement: Additional file 2: Table S2A. — Presenting enumeration of different CD45neg subgroups, as assessed by FACS, in blood samples obtained from healthy female donors, and Table S2B. presenting enumeration of different CD45neg subgroups, as assessed by DEPArray, in blood samples obtained from healthy female donors. (DOCX 90 kb) [file 13058_2016_687_MOESM2_ESM.docx]

**Supplementary Table 2A. Enumeration of different CD45^neg^ subgroups, as assessed by FACS, in blood samples obtained from healthy female donors.**

| **ID** | **CD45^NEG^ SUBGROUPS** | | |
| --- | --- | --- | --- |
|  | **E CTC** | **EM CTC** | **MES** |
|  | **E+M-*** | **E+M+*** | **E-M+*** |
| CTRL 1 | 0 | 0 | 0 |
| CTRL 2 | 0 | 0 | 0 |
| CTRL 3 | 0 | 0 | 0 |
| CTRL 4 | 0 | 0 | 0 |
| CTRL 5 | 0 | 0 | 0 |
| CTRL 6 | 0 | 0 | 0 |
| CTRL 7 | 0 | 0 | 0 |
| CTRL 8 | 0 | 0 | 0 |
| CTRL 9 | 0 | 0 | 0 |
| CTRL 10 | 0 | 0 | 0 |
| CTRL 11 | 0 | 0 | 0 |
| CTRL 12 | 0 | 0 | 0 |
| CTRL 13 | 0 | 0 | 8 |
| CTRL 14 | 0 | 0 | 15 |
| CTRL 15 | 0 | 0 | 8 |
| CTRL 16 | 0 | 0 | 0 |
| CTRL 17 | 0 | 0 | 0 |
| CTRL 18 | 0 | 0 | 0 |

*E = reactivity to the epithelial antibody cocktail. M = reactivity to the mesenchymal antibody cocktail. Results are expressed as absolute number of cells for 7.5 mL of peripheral blood.

**Supplementary Table 2B. Enumeration of different CD45^neg^ subgroups, as assessed by DEPArray, in blood samples obtained from healthy female donors.**

| **ID** | **CD45^NEG^ SUBGROUPS** | | | |
| --- | --- | --- | --- | --- |
|  | **E CTC** | **EM CTC** | **MES** | **NEG** |
|  | **E+M-*** | **E+M+*** | **E-M+*** | **E+M-*** |
| CTRL 19 | 0 | 0 | 0 | 4 |
| CTRL 20 | 0 | 0 | 0 | 2 |
| CTRL 21 | 0 | 0 | 0 | 4 |

*E = reactivity to the epithelial antibody cocktail. M = reactivity to the mesenchymal antibody cocktail. Results are expressed as absolute number of cells for 7.5 mL of peripheral blood.
