# Supplementary material for: In patients with metastatic breast cancer the identification of circulating tumor cells in epithelial-to-mesenchymal transition is associated with a poor prognosis
Source: Breast Cancer Res. 2016 Mar 9;18:30. doi: 10.1186/s13058-016-0687-3 (PMC4784394; doi:10.1186/s13058-016-0687-3)
Supplement: Additional file 3: Table S3. — Presenting the CTC count at baseline in each analyzed patient. (DOCX 18 kb) [file 13058_2016_687_MOESM3_ESM.docx]

**Supplementary Table 3. CTC count at baseline in each analyzed patient.**

| **ID** | **CTC CLASS** | | | | **GROUP***** |
| --- | --- | --- | --- | --- | --- |
|  | **E CTC** | **EM CTC** | **MES** | **NEG** |  |
|  | **E+M-*** | **E+M+*** | **E-M+*** | **E-M-*** |  |
| MAM-06 | 27 | 3 | 125 | 3 | I |
| MAM-07 | 4 | 0 | 10 | 4 | I |
| MAM-08 | 9 | 6 | 15 | 12 | I |
| MAM-09 | 3 | 0 | 69 | 12 | I |
| MAM-10 | 3 | 0 | 16 | 0 | I |
| MAM-11 | 32 | 61 | 8 | 0 | I |
| MAM-12 | 62 | 3 | 39 | 14 | I |
| MAM-14 | 10 | 0 | 14 | 0 | I |
| MAM-13 | 8 | 0 | 75 | 43 | I |
| MAM-15 | 6 | 4 | 175 | 12 | I |
| MAM-16 | 24 | 21 | 45 | 90 | I |
| MAM-18 | 37 | 15 | 29 | 14 | I |
| MAM-17 | 0 | 0 | 20 | 0 | I |
| MAM-19 | 1 | 15 | 307 | 19 | I |
| MAM-20 | 8 | 18 | 15 | 0 | I |
| MAM-21 | 4 | 0 | 6 | 6 | I |
| MAM-22 | 6 | 0 | 68 | 0 | I |
| MAM-23 | 36 | 28 | 10 | 4 | I |
| MAM-24 | 35 | 18 | 6 | 0 | I |
| MAM-25 | 9 | 3 | 6 | 117 | I |
| MAM-26 | 6 | 15 | 87 | 0 | I |
| MAM-27 | 27 | 9 | 226 | 0 | I |
| MAM-28 | 14 | 68 | 135 | 45 | I |
| MAM-29 | 4 | 0 | 64 | 0 | I |
| MAM-30 | 0 | 3 | 0 | 0 | I |
| MAM-31 | 9 | 0 | 39 | 0 | I |
| MAM-32 | 4 | 3 | 14 | 0 | I |
| MAM-33 | 6 | 4 | 41 | 9 | I |
| MAM-34 | 48 | 9 | 160 | 172 | I |
| MAM-35 | 130 | 30 | 103 | 0 | I |
| MAM-36 | 16 | 2 | 84 | 5 | I |
| MAM-37 | 33 | 0 | 96 | 9 | I |
| MAM-38 | 8 | 1 | 186 | 39 | I |
| MAM-40 | 10 | 10 | 84 | 20 | I |
| MAM-41 | 2 | 0 | 92 | 9 | I |
| MAM-42 | 15 | 0 | 129 | 6 | I |
| MAM-43 | 0 | 1 | 22 | 0 | I |
| MAM-44 | 0 | 4 | 72 | 0 | I |
| MAM-45 | 24 | 9 | 123 | 0 | I |
| MAM-46 | 1 | 0 | 3 | 0 | I |
| MAM-47 | 2 | 0 | 127 | 18 | I |
| MAM-50 | 1 | 0 | 26 | 5 | I |
| MAM-51 | 0 | 0 | 3 | 0 | I |
| MAM-52 | 4 | 4 | 94 | 2 | I |
| MAM-54 | 0 | 1 | 84 | 0 | I |
| MAM-55 | 0 | 0 | 42 | 0 | I |
| MAM-56 | 47 | 6 | 30 | 14 | I |
| *MAM-01* | *n.d.* | *n.d.* | *n.d.* | *n.d.* | *O* |
| *MAM-02* | *n.d.* | *n.d.* | *n.d.* | *n.d.* | *O* |
| *MAM-03* | *n.d.* | *n.d.* | *n.d.* | *n.d.* | *O* |
| *MAM-04* | *n.d.* | *n.d.* | *n.d.* | *n.d.* | *O* |
| *MAM-05* | *n.d.* | *n.d.* | *n.d.* | *n.d.* | *O* |
| *MAM-53* | *0* | *2* | *0* | *0* | *T* |
| *MAM-48* | *1* | *0* | *2* | *0* | *T* |
| *MAM-49* | *11* | *3* | *2* | *0* | *T* |
| *MAM-39* | *n.d.* | *n.d.* | *n.d.* | *n.d.* | *V* |

*E = reactivity to the epithelial antibody cocktail. M = reactivity to the mesenchymal antibody cocktail. Results, with exception of **, are expressed as absolute number of cells for 7.5 mL of peripheral blood.

***I = patient included in the study. V = patient excluded from the study because the volume of sampled blood was inadequate. T = patient excluded from the study because the timing of blood sampling was incorrect. O = patient excluded from the study because the blood sample was employed to optimize the technical procedures. n.d. = not determined
